# Supplementary material for: Identification of photoperiod-induced specific miRNAs in the adrenal glands of Sunite sheep (Ovis aries)
Source: Front Vet Sci. 2022 Jul 22;9:888207. doi: 10.3389/fvets.2022.888207 (PMC9354845; doi:10.3389/fvets.2022.888207)
Supplement: Supplementary file 1 [file Data_Sheet_1.ZIP › Supplementary file/miRDeep2 file.zip!/miRDeep2 file/Know_Structure/SPLP42A3/oar-mir-665.pdf]

miRBase precursor : oar-mir-665
Total read count : 11
oar-miR-665-5p read count : 1
oar-miR-665-3p read count : 9
remaining reads : 1

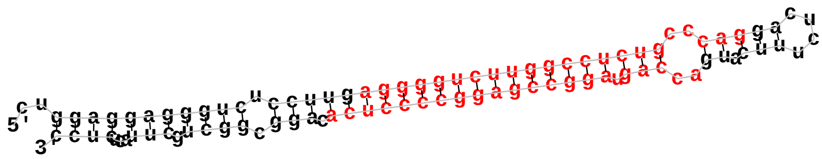

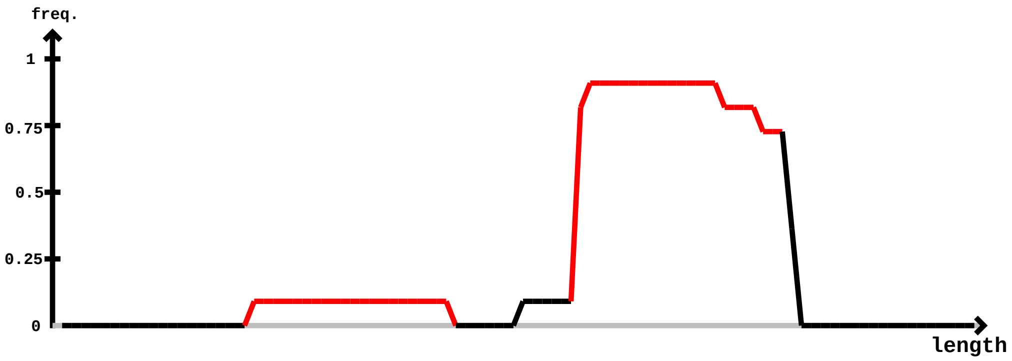


```
                                                      oar-miR-665-3p
                     oar-miR-665-5p
5'- cuggaggagggucuccuugaggggucuuggccucugcccaggacucuuucaugaccaguaggccgaggccccucacaggcggcugcuuacucucc   -3'   exp
    ..((((((((((.((((((((((((((((((((((..(((((....))).))..))).)))))))))))))))))).))).)))).))).))))..   reads   mm   sample
    ...................aggggucuuggccucugccca.......................................................   1       0    seq
    ...............................................uucaugaccaguaggccgagg...........................   1       0    seq
    .....................................................accaguaggccgaggcccc.......................   1       0    seq
    .....................................................accaguaggccgaggccccuca....................   4       0    seq
    .....................................................accaguaggccgaggccccucac...................   3       0    seq
    ......................................................ccaguaggccgaggccccucac...................   1       0    seq
```
